# Treatment of Mechanical Corneal Wounds Emergencies during the COVID-19 Pandemic: Absorbable 10-0 Vicryl (Polyglactin 910) Sutures as a Suitable Strategy

**DOI:** 10.3390/jpm12060866

**Published:** 2022-05-25

**Authors:** Nicolas Abihaidar, Gilles Thuret, Philippe Gain, Thibaud Garcin

**Affiliations:** 1 Ophthalmology Department, University Hospital, 42000 Saint-Etienne, France; nicolas.abihaidar@etu.univ-st-etienne.fr (N.A.); gilles.thuret@univ-st-etienne.fr (G.T.); philippe.gain@univ-st-etienne.fr (P.G.); 2 Institut Universitaire de France, Boulevard Saint-Michel, 75005 Paris, France; 3 Corneal Graft Biology, Engineering and Imaging Laboratory, BiiGC, EA2521, Federative Institute of Research in Sciences and Health Engineering, Faculty of Medicine, Jean Monnet University, 42000 Saint-Etienne, France

**Keywords:** corneal wound, non-penetrating corneal laceration, absorbable 10-0 Vicryl, absorbable 10-0 polyglactin 910, non-absorbable 10-0 Nylon, COVID-19 pandemic, personalized strategy, teleconsultation

## Abstract

Background—The COVID-19 pandemic has changed our standard practices: operating rooms were only available for functional emergencies and outpatient visits were drastically reduced in favor of telemedicine. Aim: To report the personalized “one-shot” surgery using absorbable 10-0 Vicryl (V10-0) or polyglactin 910 monofilament in mechanical corneal injuries from February 2020 to December 2021. Methods—Prospective case series with at least 12-months’ follow-up, in a French university hospital. Among the overall population of open or closed-globe emergencies (*n* = 40), non-penetrating corneal lamellar lacerations (long axis > 2 mm) in zone 1 (OTC group) were treated with V10-0 suture(s) (*n* = 10), replacing traditional non-absorbable 10-0 nylon suture(s) or medical options in first line. The outpatient visits were performed on day (D)10, month (M)2, M6 then every six months. One interim visit by phone teleconsultation was scheduled between D10 and M2, and other(s) as needed. The main outcome was best-corrected visual acuity (BCVA) at M6. Secondary outcomes included mainly corneal astigmatism (CA) at M6 complications. Results—Among the ten corneal wounds, there were three children (30%), eight domestic accidents (80%), three eyes with metallic foreign bodies (30%), four open-globe injuries (40%), and nine eyes that received high-speed projectiles or sharp objects (90%). The complete V10-0 suture(s) absorption occurred in all eyes between D10 and M2. At M6, mean far and near BCVA decreased from 0.680 ± 0.753 and 0.490 ± 0.338 preoperatively to 0.050 ± 0.071 and 0.220 ± 0.063 logMAR (*p* = 0.019 and *p* = 0.025 respectively), mean CA decreased from 4.82 ± 3.86 preoperatively to 1.15 ± 0.66 diopters (*p* = 0.008). BCVA and CA were unchanged thereafter. No serious adverse event nor repeated surgery occurred. The mean number of teleconsultations was 1.20 ± 0.63 without an additional nonscheduled outpatient visit. Conclusions—The absorbable V10-0 sutures might be a safe and effective alternative for eligible corneal wounds, while reducing the number of outpatient visits, especially for children (no suture removal). The COVID-19 pandemic highlighted that they are ideally suited to logistical challenges.

## 1. Introduction

Ocular trauma, and more particularly corneal trauma, is a frequent reason for ophthalmologic emergencies [1,2]. Corneal trauma can lead to permanent visual acuity deterioration, with all subsequent consequences especially in children [3]. When the corneal wound is penetrating or perforating, surgery mainly relies on non-absorbable 10-0 nylon (N10-0) sutures. This monofilament is also used for non-penetrating corneal lacerations—the most frequent pediatric eye injury [4]—but medical options such as bandage corneal lenses or biological glues exist [5]. The absorbable 10-0 Vicryl (V10-0) or polyglactin 910 sutures are less known and rarely used in traumatology. However, they offer numerous advantages, especially for pediatric cataract surgery [6].

The COVID-19 pandemic has changed our standard practices: Health Authorities in many countries, particularly in France, granted only operating room access for functional emergencies with a short-term loss of chance and reduced the number of outpatient visits in favor of telemedicine.

In this context, we tried to optimize our standard strategies for mechanical corneal wounds with a “one-shot” surgery using absorbable corneal sutures as much as possible in first-line therapy, especially for children, and promote the most suitable follow-up in times of health crisis.

We report the 12-month structural-functional results of an original surgical approach using absorbable V10-0 sutures to treat eligible mechanical corneal wounds during the COVID-19 pandemic.

## 2. Materials and Methods

We conducted a monocentric prospective study, in a university hospital (Saint-Etienne, France), approved by our local Institutional Review Board (IORG0007394, IRBN202021/CHUSTE) and conducted in accordance with the tenets of the Declaration of Helsinki. All patients were informed of the nature and intent of the study, and their written consent was collected.

Among the overall population of open or closed-globe emergencies (*n* = 40) from February 2020 to December 2021, including the French lockdowns (17 March 2020–11 May 2020, 30 October 2020–15 December 2020, and 3 April 2021–3 May 2021), we enrolled 10 eyes of 10 patients eligible for corneal sutures with absorbable V10-0 monofilament. Inclusion criteria were: traumatic mechanical injury involving either the cornea alone and/or the peripheral cornea with the limbus, i.e., zone 1 of the Ocular Trauma Classification (OTC) group [7]. We excluded the corneal wounds that required N10-0 sutures only (i.e., pure penetrating or perforating corneal wounds), an amniotic membrane graft, or that were eligible for biological glue only (long-axis wound < 2 mm) [5]. Thus, eligible mechanical corneal wounds in zone 1 (OTC group) were included as follows: non-penetrating lamellar corneal laceration—with split(s) or shredded stroma—according to Birmingham Eye Trauma Terminology (BETT) [8], with or without an intracorneal foreign body(ies), with or without corneoscleral laceration penetrating at limbus only.

Preoperative examination comprised: far and near best-corrected visual acuity (BCVA), Goldmann applanation tonometry, fundus dilated exam, slit-lamp photographs (SL-D701, Topcon, Tokyo, Japan), specular microscopy (SP2000, Topcon), anterior-segment OCT (SS-OCT CASIA2, Tomey, Nürnberg, Germany), macular OCT (Spectralis, Heidelberg Engineering, Heidelberg, Germany), fundus retinography (CR2-AF, Canon, Tokyo, Japan) and head-neck imaging as needed.

All surgical procedures were standardized and performed under general anesthesia by one experienced surgeon (T.G): absorbable V10-0 (Ethicon, Raritan, NJ, USA) sutures for eligible corneal wounds, and N10-0 (Alcon, Geneva, Switzerland) sutures only for the opened sclerocorneal zone at the limbus when present. All patients had one overnight hospital stay. Postoperative treatment combined topical anti-inflammatory and antibiotics, lubricating drops at will. Outpatient visits were performed examination at day (D)10, month (M)2, which is the average expected time of complete suture(s) absorption [9], M6, then every six months. A scheduled phone teleconsultation took place between D10 and M2. Interim visits were as needed: teleconsultation(s) were favored, and the need for an outpatient visit(s) was based on the teleconsultation outcome.

The primary outcome was BCVA at M6. Visual improvement was defined as an increase of at least 0.3 logarithms of the minimal angle resolution (logMAR) units (equivalent to a 15-letter change on the ETDRS chart). Secondary outcomes included mean corneal astigmatism (CA)—measured by the mean of auto-refractometer data (ARK-1s, NIDEK) and axial topography data (SS-OCT CASIA2)—at M6, number of interim visits, and potential complications.

The normality of continuous data distribution was analyzed with the Shapiro-Wilk test. Normally distributed data were described by their mean ± standard deviation (SD). When the variable followed a normal distribution, a paired Student *t*-test was used to compare measurements during the follow-up. Rejection of the null hypothesis was defined as α < 0.05 (*p* two-tailed). The Holm-Sidak method was used when multiple comparisons occurred. We used SPSS 25.0 (IBM, Armonk, NY, USA).

## 3. Results

### 3.1. Baseline Data

Among 40 open or closed-globe surgical emergencies, 10 corneal wounds of 10 patients were analyzed with a mean follow-up of 15.0 ± 3.2 months (range, 12–18). Table 1 gives the demographic data and characteristics of the eyes. Corneal lacerations were mainly associated with high-speed projectiles or sharp objects (*n* = 9, 90%), and other else blunt contusions occurred. Accidents were mainly domestic (*n* = 8, 80%), and concerned adults (*n* = 7, 70%) and males (sex ratio 4.0). All eyes were phakic without patent cataracts. All corneal wounds presented a mean long-axis 6.2 ± 3.0 mm (range, 2.8–11) with a spiroid profile in zone 1 (OTC group), and were treated by V10-0 sutures, with a mean number of 4.8 ± 3.8 sutures (range, 1–14). Only three corneal wounds had multiple splits. The average maximum depth of the lamellar laceration (non-penetrating wounds), measured by anterior-segment OCT, was 55 ± 12.2% (range 40–70) of total corneal thickness.

Mean baseline data were age 31.5 ± 20.8 years (range, 5–65); far BCVA 0.680 ± 0.753 logMAR (range, 0.0–2.0); near BCVA 0.490 ± 0.338 logMAR (range, 0.2–0.9); CA 4.82 ± 3.86 diopters (range, 0.3–12.2); intraocular pressure (IOP) 11.4 ± 2.8 mmHg without history of glaucoma (range, 8–16); and ocular trauma score (OTS) [10] 3.5 ± 1.1 (range 2–5).

### 3.2. Primary and Secondary Outcomes Results

At M6, compared to baseline, mean far BCVA decreased to 0.050 ± 0.071 logMAR (*p* = 0.019) and mean near BCVA decreased to 0.220 ± 0.063 logMAR (*p* = 0.025), and mean CA decreased to 1.15 ± 0.66 (*p* = 0.008). As defined by our criteria, far BCVA improved in 10 eyes (100%), as did near BCVA. These findings remained unchanged thereafter.

**Figure 1:**
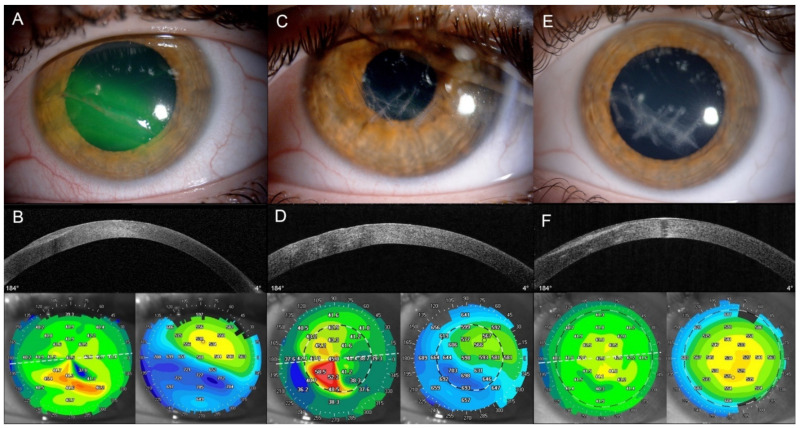
Non-penetrating corneal laceration caused by scissors, on a 12-year-old child. Multimodal imaging of the corneal wound. ((**A**,**C**,**E**): Slit lamp images (Topcon). (**B**,**D**,**F**): Anterior segment OCT (AS-OCT), topography and pachymetry map (SS-OCT CASIA 2, Tomey) images). (**A**,**B**) Baseline examination showing a 9-mm spiroid linear laceration in zone 1 (OTC group), with some rust particles in the shredded stromal bed. B-scan showed wound spiroid wound interface (hyperreflective line), in the anterior third of the corneal thickness. Local variations occurred along the wound on topography (corneal astigmatism 2.3 diopters) and reactional stromal edema of the lower hemi-cornea (+100 to 150 µm versus upper part) were highlighted on the pachymetry map. Maximum lamellar depth was 40% of total corneal thickness. Preoperative far visual acuity (VA) was 20/50 (0.4 logarithm of the minimal angle resolution (logMAR)) and near VA was Jaeger 7 (0.5 logMAR). (**C**,**D**) Postoperative result on Day 10, showing seven light-purple threads of polyglactin 910 (or V10-0), which spared the visual axis. Inferior hemi-cornea was clearer with normal conjunctiva, no local inflammation (surface or anterior chamber) was noted. Comparative B-scan showed anterior stromal hyperreflective lines corresponding to the treads. Moderate transient suture-induced astigmatism was visible on topography, and stromal edema reduced with a more homogeneous pachymetry map. (**E**,**F**) Mid-term result at Month 2, the wound has healed with a longitudinal opacity corresponding to the initial wound interface. No more threads were present. A mild loss of transparency was present along the initial threads path due to suture absorption. Comparative B-scan showed longitudinal anterior stromal hyperreflectivity corresponding to the initial spiroid flap with secondary reorganized keratocytes, and the suture path was still visible more posterior transversely (small hyperreflective lines with a mild condensation of the posterior stroma). There was no residual surgically induced astigmatism (1.3 diopters, similar to the contralateral healthy eye). Pachymetry map was homogeneous and had a normal profile. Postoperative far VA was 20/25 (0.1 logMAR) and near VA was Jaeger 2 (0.2 logMAR).

**Figure 2:**
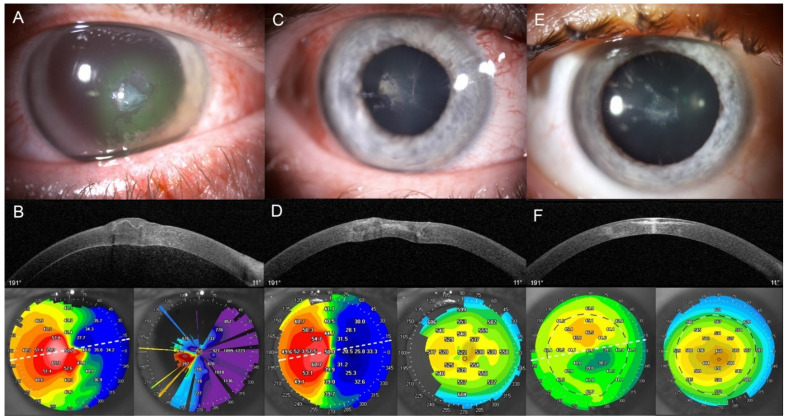
Disorganized central laceration in stromal mush, caused by high-speed projectile (stone) while riding a motorcycle, on a 43-year-old man. Multimodal imaging of the corneal wound. [(**A**,**C**,**E**): Slit lamp images (Topcon). (**B**,**D**,**F**): Anterior segment OCT (AS-OCT), topography and pachymetry map (SS-OCT CASIA 2, Tomey) images]. (**A**,**B**) Baseline examination showing a central wound of 2.75 mm in diameter in zone 1 (OTC group), with epithelial defect, stromal edema and loss of transparency due to a mush under the retracted Bowman’s membrane (BM) with inferonasal hinge. A second laceration was close to the inferior limbus. Anterior chamber was normal. B-scan showed the disorganized anterior stroma with the BM flap (waved hyperreflective line). There were 12.2 diopters of corneal astigmatism, with major artifacts (suboptimal segmentations not improvable of software) on topography (white dashed line indicates B-scan axis) and pachymetry maps. Maximum lamellar depth was 47% of total corneal thickness. Preoperative far visual acuity (VA) was 20/2000 (2.0 logarithm of the minimal angle resolution (logMAR)). (**C**,**D**) Postoperative result at Day 10, showing four central light-purple threads of polyglactin 910 (or V10-0) which spared the visual axis and stretched the BM flap, and three peripheral V10-0 threads which treated spiroid paralimbic scalp. There was a very significant improvement of central corneal transparency. B-scan showed progressive reorganization of the anterior stroma with a BM flap flattened thanks to suture-induced tension. Artifacts were reduced on topography with transient suture-induced astigmatism. Initial post-traumatic stromal mush reduced central corneal thickness. (**E**,**F**) Mid-term result at Month 2, the wound healed with a mild central opacity corresponding to the interface of wounded BM. No more threads were present. A mild loss of transparency was visible at the entry and exit points of the threads path. Comparative B-scan showed longitudinal anterior stromal hyperreflectivity corresponding to the wounded BM, which was flattened thanks to surgery, but thickened with reorganized keratocytes. Residual astigmatism was 1.9 diopters. Pachymetry map was homogeneous and central corneal thickness was reduced in the area of the initial wound (central 3 mm). Postoperative far VA was 20/32 (0.2 logMAR) and near VA was Jaeger 5 (0.4 logMAR).

Typical examples of follow-up are detailed in Figure 1, which shows a linear 9-mm corneal wound from the children subgroup, and in Figure 2, which shows a central corneal wound with shredded stroma from the adults’ subgroup.

Complete V10-0 sutures absorption was achieved in all eyes at M2 without complication, especially with no neovascularization, inflammation, allergy, or loosening of the knot. All corneal wound interfaces presented a relative loss of transparency. No adverse events occurred, particularly infectious (abscesses or endophthalmitis), nor was any additional surgery needed. Compared to baseline, no significant IOP variation was observed at D10 (*p* = 0.067), M2 (*p* = 0.342), M6 (*p* = 0.431), or M12 (*p* = 0.109). Specular endothelial cell density did not change significantly between D10 (3068 ± 520 cell/mm^2^) and M12 (2944 ± 568 cell/mm^2^) (*p* = 0.552).

The mean number of teleconsultations was 1.20 ± 0.63 (range, 1–3) without interim nonscheduled outpatient visits: only one patient needed two teleconsultations after M2.

## 4. Discussion

The “one-shot” surgery using V10-0 sutures achieved encouraging structural-functional results for eligible mechanical corneal wounds during the COVID-19 pandemic: significant vision and CA improvement occurred in all eyes. Baseline characteristics of patients (young age) and selected corneal wounds (type, zone 1 OTC group) have been known to be factors of good outcome [11]. At M6, we had excellent outcomes thanks to our personalized strategy: All eyes (100%) had BCVA ≥20/40 (logMAR ≥ 0.3), compared to the estimated probability between 44% and 74% according to the baseline mean OTS score [10] of our series. We reported no complications, especially no V10-0 sutures-related complications contrary to Bainbridge et al. [9] (broken suture, partial absorption with mild corneal haze).

Unlike medical options available for similar wounds, our personalized “one-shot” surgery using the V10-0 sutures strategy allowed efficient and safe telemedicine [12,13] without multi-weekly early outpatient visits. We were only able to propose this strategy because these eligible wounds had an initial better visual prognosis and were less likely to cause infectious and anatomical complications than penetrating corneal wounds [14,15]. Our personalized follow-up was suitable for the Health Authorities’ guidelines. The only patient who had two interim teleconsultations presented an anxious personality.

As reported before, the young adult [16] males [17,18] were the most represented, as were the domestic accidents due to the COVID-19 pandemic including the lockdowns.

The V10-0 sutures seemed particularly appropriate to treat non-penetrating corneal lacerations in zone 1 (OTC group) in first-line therapy, for mutually non-exclusive reasons: 1) The properties of the polyglactin 910 has shown advantages since the seventies [19]: mainly in pediatric cataract surgery [6] avoiding suture removal, and more marginally in adults cataract surgery [9], or in combined glaucoma surgery [20,21,22] reducing the risk of early over-filtration and preserving filtering bleb without additional inflammation. Its characteristics have only been studied on simple linear surgical incisions and never to our knowledge in mechanical corneal wounds. V10-0 absorption occurs by hydrolysis alone making it invisible [9]. 2) Medical options such as bandage corneal lenses or biological glues exist [5] for corneal lacerations, but they were not suitable in times of health crisis: they would require a long and broad-spectrum topical antibioprophylaxis covering both gram-positive cocci and gram-negative bacilli, and recurrent outpatient visits. Many complications—bacterial keratitis, sterile corneal infiltrates, transient corneal edema, peripheral or paracentral corneal neovascularization—can also occur [5,23]. Besides, post-traumatic CA could be irregular and less predictable, with a more severe dry eye disease. 3) When the corneal wound has a long-axis >2 mm, sutures may improve structural-functional recovery. Barr et al. [14] indeed correlated the length of a corneal wound with the amount of CA and with a poor visual outcome. The N10-0, recommended treatment in penetrating wounds, has useful known mechanical properties [24,25]: hypoantigenicity, elasticity, and durable tensile strength over time. However, in the case of corneal lacerations, V10-0 presents specific technical advantages with a spatulated needle [19]: it can be more accurate and easier to handle through the shredded stroma, cutting less tissue when tightening. We used it when the residual stromal bed was greater than ⅓ of the total corneal thickness, which ensures safe recovery of corneal biomechanics. 4) The initial cost was comparable between N10-0 and V10-0 use: the public price per unit was 20,08 and 19,84 euros respectively; the remaining (hospital stay) expenses were comparable. However, the global cost was in favor of V10-0: the absorption avoided suture removal—particularly for the children avoiding additional surgery—and additional early outpatient visits while allowing phone teleconsultation(s), thus it was a cost-saving strategy [13,26]. These logistical advantages were put to use during the pandemic.

Our study had several strengths: a prospective follow-up of at least 1-year, baseline homogeneity of eye injuries, and the use of standardized definitions, surgery, and imaging. Its limitations: this was a monocentric, non-controlled, non-randomized small pilot study. Larger numbers with longer follow-up, ideally in a randomized multicentric study (including a precise ancillary cost-effectiveness evaluation) comparing V10-0 sutures with medical treatments and with N10-0, are needed to assess V10-0′ use in the surgical management of corneal lamellar lacerations and to potentially extend its scope in corneal trauma. This reported strategy will be required to demonstrate the relevance of its advantages, especially logistics, beyond the pandemic context in which it was studied.

## 5. Conclusions

The absorbable V10-0 seems to appear as a safe and effective option to treat eligible mechanical non-penetrating corneal lamellar lacerations, with numerous advantages, especially in times of health crisis.

## Figures and Tables

**Table 1:** Demographic data and preoperative characteristics of eyes (*n* = 10).

Characteristic		Number (%)
Patient	Child	3 (30%)
	Adult	7 (70%)
Accident	Domestic	8 (80%)
	Workplace	2 (20%)
Injury	With foreign body	3 (30%)
	Closed-globe	6 (60%)
	Open-globe	4 (40%)
Suture Type	10-0 Vicryl only	6 (60%)
	10-0 Vicryl and 10-0 nylon ^#^	4 (40%)

# 10-0 nylon sutures were only used when there was an opened sclera or cornea at the limbus, other else corneal sutures were done with 10-0 Vicryl.

## Data Availability

The datasets used and/or analyzed during the current study are available from NA or TG on reasonable request.

## References

[1] Wilson M.R., Wooten F., Williams J. (1991). Frequency and characteristics of ocular trauma in an urban population. J. Natl. Med. Assoc..

[2] Négrel A.-D., Thylefors B. (1998). The global impact of eye injuries. Ophthalmic Epidemiol..

[3] Vora G.K., Haddadin R., Chodosh J. (2013). Management of Corneal Lacerations and Perforations. Int. Ophthalmol. Clin..

[4] Liu M.-L., Chang Y.-S., Tseng S.-H., Cheng H.-C., Huang F.-C., Shih M.-H., Hsu S.-M., Kuo P.-H. (2010). Major Pediatric Ocular Trauma in Taiwan. J. Pediatr. Ophthalmol. Strabismus.

[5] Macsai M.S. (2000). The management of corneal trauma: Advances in the past twenty-five years. Cornea.

[6] Matalia J., Panmand P., Ghalla P. (2018). Comparative analysis of non-absorbable 10-0 nylon sutures with absorbable 10-0 Vicryl sutures in pediatric cataract surgery. Indian J. Ophthalmol..

[7] Pieramici D.J., Sternberg P., Aaberg T.M., Bridges W.Z., Capone A., Cardillo J.A., De Juan E., Kuhn F., Meredith T.A., Mieler W.F. (1997). A System for Classifying Mechanical Injuries of the Eye (Globe). The Ocular Trauma Classification Group. Am. J. Ophthalmol..

[8] Kuhn F., Morris R., Witherspoon C. (2002). Birmingham Eye Trauma Terminology (BETT): Terminology and classification of mechanical eye injuries. Ophthalmol. Clin. N. Am..

[9] Bainbridge J.W., Teimory M., Kirwan J.F., Rostron C.K. (1998). A prospective controlled study of a 10/0 absorbable polyglactin suture for corneal incision phacoemulsification. Eye.

[10] Kuhn F., Maisiak R., Mann L., Mester V., Morris R., Witherspoon C.D. (2002). The Ocular Trauma Score (OTS). Ophthalmol. Clin. N. Am..

[11] Kuhn F., Morris R., Witherspoon C., Mann L. (2006). Epidemiology of Blinding Trauma in the United States Eye Injury Registry. Ophthalmic Epidemiol..

[12] Saleem S.M., Pasquale L.R., Sidoti P.A., Tsai J.C. (2020). Virtual Ophthalmology: Telemedicine in a COVID-19 Era. Am. J. Ophthalmol..

[13] Etges A.P.B.D.S., Zanotto B.S., Ruschel K.B., da Silva R.S., Oliveira M., Moreira T.D.C., Cabral F.C., de Araujo A.L., Umpierre R.N., Gonçalves M.R. (2022). Telemedicine Versus Face-to-Face Care in Ophthalmology: Costs and Utility Measures in a Real-World Setting. Value Health Reg. Issues.

[14] Barr C.C. (1983). Prognostic Factors in Corneoscleral Lacerations. Arch. Ophthalmol..

[15] Agrawal R., Rao G., Naigaonkar R., Ou X., Desai S. (2011). Prognostic factors for vision outcome after surgical repair of open globe injuries. Indian J. Ophthalmol..

[16] Liggett P.E., Pince K.J., Barlow W., Ragen M., Ryan S.J. (1990). Ocular Trauma in an Urban Population. Review of 1132 cases. Ophthalmology.

[17] Thylefors B. (1992). Epidemiological patterns of ocular trauma. Aust. N. Z. J. Ophthalmol..

[18] Cassen J.H. (1997). Ocular trauma. Hawaii Med. J..

[19] Bartholomew R.S., Phillips C.I., Munton C.G. (1976). Vicryl (polyglactin 910) in cataract surgery. A controlled trial. Br. J. Ophthalmol..

[20] Vyas A.V., Bacon P.J., Percival S.P.B. (1999). The benefits of phacotrabeculectomy using 10-0 polyglactin sutures. Eye.

[21] Raina U.K., Tuli D., Mehta D.K. (1999). Polyglactin sutures versus nylon sutures for scleral flap suturing in trabeculectomy. Ophthalmic Surg. Lasers.

[22] Rayees A.S., Prem C.K., Viney G. (2021). Trabeculectomy: Is releasable suture trabeculectomy a cause of better bleb?. Rom. J. Ophthalmol..

[23] Donnenfeld E.D., Selkin B.A., Perry H.D., Moadel K., Selkin G.T., Cohen A.J., Sperber L.T. (1995). Controlled Evaluation of a Bandage Contact Lens and a Topical Nonsteroidal Anti-inflammatory Drug in Treating Traumatic Corneal Abrasions. Ophthalmology.

[24] Rennie L., Fleming W., Clark D., Ellerton C., Bosanquet R. (1994). Some mechanical properties of 10/0 monofilament nylon sutures. Eye.

[25] Potvin R., Matossian C., Makari S. (2015). Cataract surgery and methods of wound closure: A review. Clin. Ophthalmol..

[26] Parikh D., Armstrong G., Liou V., Husain D. (2020). Advances in Telemedicine in Ophthalmology. Semin. Ophthalmol..

